# Restoring T Cell Homeostasis After Allogeneic Stem Cell Transplantation; Principal Limitations and Future Challenges

**DOI:** 10.3389/fimmu.2018.01237

**Published:** 2018-06-18

**Authors:** Moutuaata M. Moutuou, Gabriel Pagé, Intesar Zaid, Sylvie Lesage, Martin Guimond

**Affiliations:** ^1^Division d’Hématologie-Oncologie, Centre de Recherche de l’Hôpital Maisonneuve-Rosemont, Montréal, QC, Canada; ^2^Département de Microbiologie, Infectiologie et Immunologie, Université of Montréal, Montréal, QC, Canada

**Keywords:** interleukin-7, dendritic cells, lymphopenia, lymphocytes, stem cell transplantation, GVHD, IL-7, SDF-1α

## Abstract

For several leukemia patients, allogeneic stem cell transplantation (allogeneic-SCT) is the unique therapeutic modality that could potentially cure their disease. Despite significant progress made in clinical management of allogeneic-SCT, acute graft-versus-host disease (aGVHD) and infectious complications remain the second and third cause of death after disease recurrence. Clinical options to restore immunocompetence after allogeneic-SCT are very limited as studies have raised awareness about the safety with regards to graft-versus-host disease (GVHD). Preclinical works are now focusing on strategies to improve thymic functions and to restore the peripheral niche that have been damaged by alloreactive T cells. In this mini review, we will provide a brief overview about the adverse effects of GVHD on the thymus and the peripheral niche and the resulting negative outcome on peripheral T cell homeostasis. Finally, we will discuss the potential relevance of coordinating our studies on thymic rejuvenation and improvement of the peripheral lymphoid niche to achieve optimal T cell regeneration in GVHD patients.

## Key Points

- GVHD effect on the thymus.
- GVHD effect on the peripheral niche.
- Addressing the dysfunction of the thymus and the peripheral niche to improve T cell regeneration in GVHD patients.

## Introduction

Allogeneic-SCT was developed to treat leukemia and lymphoma as well as congenital or acquired hematologic conditions. Despite important side effects, allogeneic-SCT remains the only curative treatment for several patients with high risk refractory hematologic cancers. Acute graft-versus-host disease (aGVHD) is a serious complication of allogeneic-SCT that occurs when donor lymphocytes react to normal host-tissues. Paradoxically, alloreactive T cells can improve survival by eliminating residual leukemia cells that survive SCT; a reaction known as graft-versus-leukemia effect (1). Following SCT, donor hematopoietic stem cells home to the bone marrow (BM) and differentiate into white cells, megakaryocytes and erythrocytes. While the recovery of innate immune cells is relatively fast, regeneration of lymphocytes is slower and can be further delayed by aGVHD (2, 3). For several years, it was believed that the failure to recover T cells post-SCT was essentially attributed to thymic dysfunction. Today, it is well known that the size of the lymphocyte pool is controlled by the thymus but also by the peripheral niche that provides resources for T cells to survive in the periphery. Recent functional studies have demonstrated a significant reduction in the bioavailability of peripheral T cell resources in graft-versus-host disease (GVHD) hosts, which contributes to the more important immunosuppression in allogeneic-SCT compared with autologous-SCT. As a result, lymphopenia is typically more severe in GVHD patients and clinical options to accelerate lymphocyte reconstitution are virtually inexistent because of the risk to aggravate aGVHD (4, 5). In this mini-review, we will address how aGVHD affects thymopoiesis and the peripheral niche, and we will propose potential strategies to improve immune reconstitution after allogeneic-SCT.

## Immune Reconstitution in a Non-GVHD Setting

**Figure 1:**
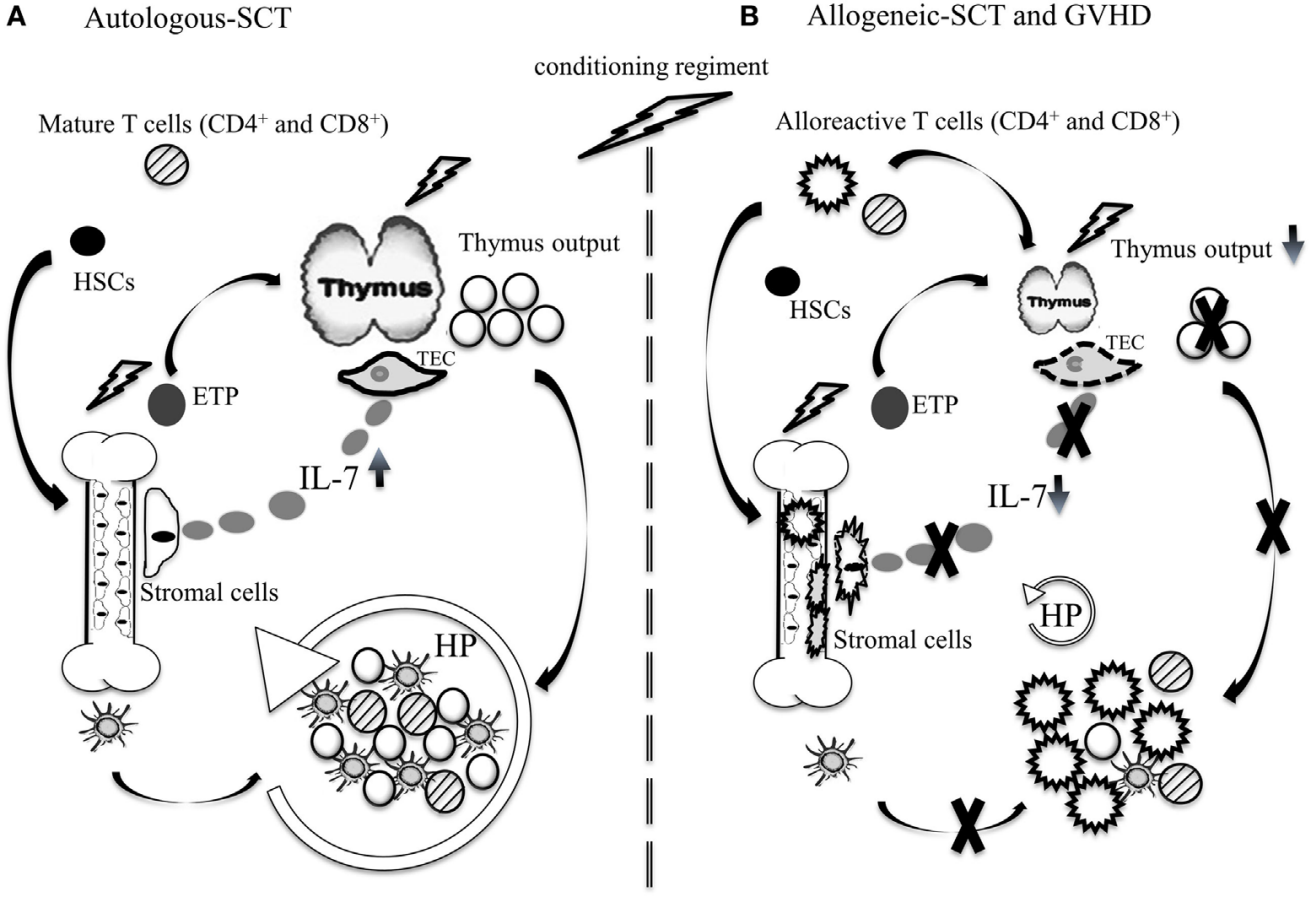
Immune reconstitution after autologous and allogeneic-SCT. **(A)** Autologous-SCT: chemotherapeutic insults affect the BM and thymopoiesis. During this period, thymopoiesis is inefficient and T cell regeneration occurs primarily through HP of mature T cells contained in the graft. The production of DCs occurs relatively early after autologous SCT and combined with elevated systemic IL-7, produced by stromal cell of primary and secondary lymphoid organs, they induce HP of mature T cells. In younger patients, rapid thymopoiesis recovery contributes to normalize CD4^+^ T cell counts and T cell receptor diversity. **(B)** Allogeneic-SCT: the combined GVHD and chemotherapeutic insults to the thymus and the BM induce long-lasting dysfunction of thymopoiesis and the peripheral lymphoid niche. Damages to the BM microenvironment are mediated primarily by alloreactive CD4^+^ T cells. During GVHD, DC production is reduced and systemic IL-7 is low, which constrain HP of non-alloreactive naïve T cells. Depending on the severity of GVHD and patient’s age, the dysfunction of the thymus can persist for several years.

Lymphocyte regeneration can occur through thymopoiesis and/or *via* homeostatic proliferation (HP) of mature lymphocytes (2). In younger patients, thymic regeneration typically occurs during the first-year post-transplantation and is normally followed by rapid normalization of T cell counts (2). In adults, age-related thymic involution combined with therapy-induced cytotoxic insults result in prolonged thymic dysfunction. During this period, T cell regeneration occurs primarily through HP of mature lymphocytes contained in the graft (Figure 1A). In addition to interleukin-7 (IL-7), T cell receptor (TCR) stimulation by major histocompatibility complexes (MHCs) class I or II is necessary for HP of CD8^+^ and CD4^+^ lymphocytes, respectively (6). While HP is sufficient for restoring CD8 counts, it is normally insufficient for CD4^+^ T lymphocytes and the full recovery of the CD4 subset can take several months or years to occur and depends largely on thymic recovery (2). B cell recovery takes between 3 and 6 months to occur (7, 8), whereas DC recovery after autologous-SCT is normally fast. Given that DCs are important for NK cell homeostasis, they likely influenced NK regeneration which also occurs within few weeks post-SCT (9, 10) (Table 1).

**Table 1:** Time line of immune reconstitution of immune cells after autologous and allogeneic-SCT (7, 11–17).

Cells subsets	Autologous-SCT	Allogeneic-SCT (years)
CD4^+^ lymphocytes	>1 year	>2
CD8^+^ lymphocytes	1–3 months	1–2
NK cells	1–2 months	1–2
Dendritic cells	1–2 months	1–2
B lymphocytes	3–6 months	>2

## Immune Reconstitution after Allogeneic-SCT and GVHD

The immunosuppression that occurs after allogeneic-SCT is typically more important than the level of immunosuppression normally seen after autologous-SCT. Patients undergoing allogeneic-SCT experience a phase of profound lymphopenia that can last several months or years (18, 19). Depending on the severity of the aGVHD, the regeneration of both CD4^+^ and CD8^+^ lymphocytes can be further delayed. The current models put forth to explain how aGVHD affects T cell reconstitution relates to two primary factors: GVHD-mediated damage to the thymic microenvironment essential for T cell production (20); and the dysfunction of the peripheral niche essential for the survival and HP of naïve CD4^+^ and CD8^+^ T lymphocytes in the periphery (Figure 1B) (21–23). These animal studies have provided a new model to explain the profound immunosuppression typically seen in GVHD patients.

**Figure 2:**
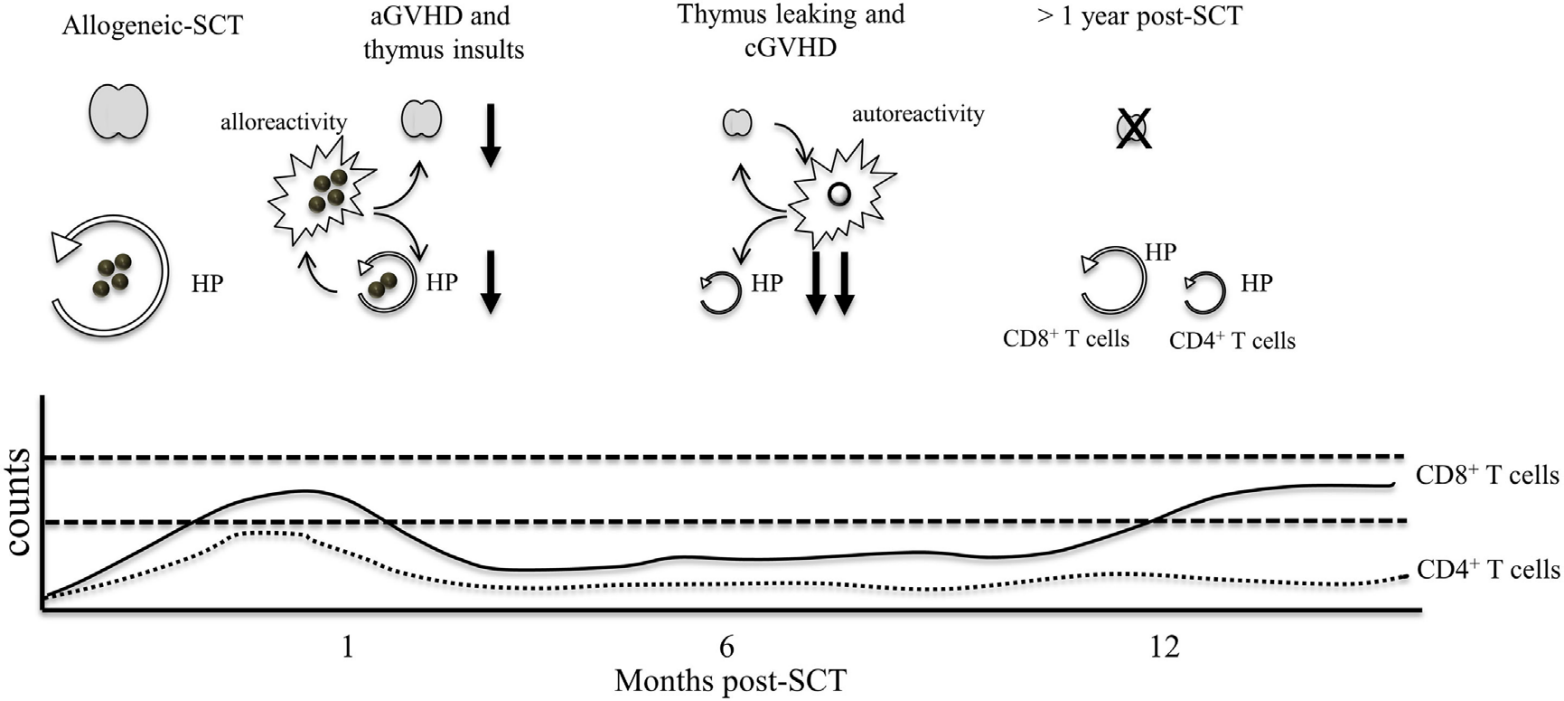
The effect of GVHD on lymphocyte numbers. Following chemotherapy, thymic insults induce thymic involution and loss of thymic output. Early after T cell infusion, CD4^+^ and CD8^+^ T cell counts increase as a consequence of HP and alloreactive T cell activation. At 1 month post-allogeneic-SCT, GVHD T cells induce damage to the thymus and the peripheral niche, resulting in severe thymic dysfunction and lower HP of T cells. During this period, patients are profoundly lymphopenic. After several months, the thymus may leak few autoreactive T cells, which contribute to the development of cGVHD. Autoreactive T cells can further damage the thymus and the peripheral niche, resulting in long lasting immunosuppression. At 1 year post-GVHD, rises in CD8 counts can occur through HP whereas HP of CD4^+^ T cells remains highly inefficient resulting in chronic CD4 lymphopenia that can persist for several years.

In contrast, the effect of chronic GVHD (cGVHD) on T cell regeneration is not as well understood. cGVHD occurs normally after aGVHD and during this period, T cell regeneration is already compromised. While aGVHD is mediated by mature lymphocytes contained in the graft, the origin of cGVHD appears related to leakage and release of donor-derived autoreactive lymphocytes by the thymus (Figure 2). As a result, clinical manifestations are different from aGVHD with cGVHD symptoms resembling those in patients with systemic autoimmune diseases (24).

Immunosuppressive therapies use to control GVHD can affect lymphocyte reconstitution. Cyclosporine A and methotrexate alter B cell differentiation and thymocytes and peripheral T cell survival by interfering with TCR signaling (7, 25, 26). Novel drugs used to control refractory cGVHD are tyrosine kinase inhibitors, which can interfere with TCR or IL-7 signaling to reduce lymphocyte activation with potential detrimental effects on T cell survival (27–29). Thus, in addition to chemotherapeutic and GVHD insults that affect lymphoid organs, the prophylaxis used to control GVHD can further constrain lymphocyte regeneration after allogeneic-SCT.

## GVHD Effects on the Thymus

The rich microenvironment of the thymus in MHC-I and II expression by thymic epithelial cells (TECs) and antigen-presenting cells render the thymus a significant target for alloreactive lymphocytes (30). While studies have demonstrated that the thymic recovery that occurs after autologous-SCT is associated with rapid normalization of the T cell repertoire (2), thymic rebound that occurs after allogeneic-SCT is often insufficient for restoring T cell counts and diversity, even in children (31, 32). Such differences in TCR diversity following autologous and allogeneic-SCT suggest that some elements of the thymus undergo significant damages by alloreactivity. Indeed, thymic-dependent immune reconstitution of T lymphocytes is severely impaired after allogeneic-SCT and further compromised by aGVHD (20, 33). The contribution of cGVHD T cells to thymic insults remains largely unknown since thymus atrophy is normally severe during this period. However, thymus infiltration by autoreactive T cells can occur in mice with systemic autoimmune diseases, suggesting a potential contribution of cGVHD T cells to thymic damage (34).

Evaluation of thymopoiesis can be measured by T-cell receptor excision circles (TREC) that are generated from the rearrangement of the V and J segments at the TCRα locus and thus correlate with thymic output of naïve T cells (35, 36). Depending of the robustness of the thymic output before transplantation, clinical outcomes and incidence of GVHD may vary (37). During GVHD, TREC levels are normally low and correlate with low levels of recent thymic emigrants (RTEs) detected in the blood (36, 38–41). Other factors such as age, preparative regimen, source of stem cells could also affect thymopoiesis (42, 43).

Normal thymopoiesis proceeds in a thymic microenvironment composed of a cortex and a medulla separated by the cortico–medullary junction. TECs are the major component of the thymus stroma that guide thymocyte development. Cortical thymic epithelial cells (cTECs) and medullary thymic epithelial cells (mTECs) have distinct functions and play a critical role during positive and negative selection of thymocytes to produce conventional T cells and non-conventional FOXP3^+^ regulatory CD4^+^ T cells (44–48). Following allogeneic-SCT, aGVHD induces loss of the delimitation between the cortex and the medulla (49, 50). Alloreactive lymphocytes can eliminate cTECs and mTECs, which explains the reduction of the number of mTECs expressing the transcription factor AIRE “autoimmune regulator” during GVHD (51–53). Loss of TECs is associated with diminished IL-7 production, which contributes to thymocyte death and loss of thymic output during aGVHD (54). While GVHD insults to cTECs can impair positive selection, GVHD alteration to mTECs is more insidious and could impair the negative selection of autoreactive thymocytes and contribute to cGVHD (55, 56). Furthermore, several DC subsets collaborate with mTECs to induce negative selection of self-reactive thymocytes and during GVHD, they are eliminated by alloreactive T cells, which lead to the development of cGVHD (57–60). Thus, although thymic recovery is an essential step toward normalization of T cell counts, the production of self-reactive T cells during/after aGVHD could negatively impact the benefit of thymic dependent T cell regeneration. Effective thymic recovery in a GVHD setting will need to include means to ensure adequate positive and negative selection of thymocytes to preclude the generation of potentially autoreactive lymphocytes that could exacerbate the pathology.

## GVHD Effects on the Peripheral Niche

Several studies have demonstrated that GVHD induces significant damage to the peripheral niche that controls T cell homeostasis (21–23). During lymphopenia, IL-7 accumulates due to a reduced consumption by the limited number of T cells (61). In addition, T cells have increased accessibility to MHC-expressing DCs and the increase in both IL-7 and MHC accessibility promotes T lymphocyte HP (61). During GVHD, however, the lymphopenic environment is strikingly different and survival and HP of T cells are impaired (23, 62–64). For one, alloreactivity induces damages to stromal cells of primary and secondary lymphoid organs, the primary source of IL-7 (22, 65–67). In addition, DC regeneration is decreased in GVHD (23). CD4^+^ T cell HP depends on MHC-II presentation by DCs, such that this effect alone could explain the lack of CD4 regeneration. However, it does not necessarily explain lack of CD8^+^ T cell HP, as MHC-I is ubiquitously expressed, and CD8 HP is probably not exclusively dependent on MHC-I expressed by DCs. Additional studies are needed to identify elements of the peripheral niche that limit CD8 recovery in GVHD hosts.

Several studies have demonstrated that DC numbers are reduced during GVHD and depletion/inactivation of recipient DCs before allogeneic-SCT can reduce GVHD (63, 68–71). The mechanism preventing DC regeneration during GVHD is not fully understood (23, 64, 72). However, two potential mechanisms have been proposed to explain diminished DC counts in GVHD hosts: the elimination of DCs by alloreactive lymphocytes and the disruption of the BM microenvironment that constrains DC production. Indeed, T cells can eliminate DCs after priming (73–75) and following allogeneic-SCT, recipient and donor-derived DCs can be eliminated by alloreactive lymphocytes (23). However, the long-lasting DC depletion that occurs during GVHD is unlikely only due to the allo-immune response against mature DCs since the elimination of alloreactive lymphocytes post-GVHD does not restore DC counts.

Damage to the BM microenvironment can induce myelosuppression after allogeneic-SCT. BM stromal cells provide growth factors such as GM-CSF, M-CSF, and Flt3-L, which are essential for HSC differentiation into mature cells. However, little is known about how these factors are affected by GVHD in the BM. While serum analysis can provide some insight about variation of these factors, the evaluation of BM biopsy is probably more accurate since several of these factors are also produced by immune cells (76). For example, Flt3-L levels in the BM differ from those seen in the blood of GVHD mice (23). The retention/egress of BM cells is also important for the differentiation of DCs. Stromal derived factor-1 alpha (SDF-1α) regulates integrin expression on HSCs and during GVHD, SDF-1α is diminished (23). Low SDF-1α levels inside the BM likely explain the accumulation of poorly differentiated DC precursors found in the blood during GVHD (64). The low SDF-1α levels in the BM are line with the low documented B cell levels in GVHD (77). Thus, access to BM specimens is essential to define the factors that limit DC regeneration necessary for maintaining both T and B cell homeostasis.

## Restoring Thymopoiesis

Thymus rejuvenation represents the best option to restore T cell repertoire diversity in transplanted patients. Today, the adoptive transfer of T cell precursors and the transplantation of thymic tissues are two interesting options to achieve this goal. The administration of T cell precursors has demonstrated its efficacy at correcting intrinsic defects to common lymphoid progenitors (CLPs) to improve thymopoiesis. However, these studies were performed in a non-GVHD setting and although CLPs are decreased during GVHD (23), it is unclear whether the administration of T cell precursors could improve thymopoiesis if the thymic epithelium has been damaged by alloreactive lymphocytes. Nonetheless, interaction between T cell precursors and TECs may help TECs to recover post-GVHD (78, 79). The administration of keratinocyte growth factor (KGF) protects the thymic epithelium from GVHD insults in mice (80). In humans, however, KGF (Palifermin) has been tested for CD4^+^ T cell recovery in HIV patients but the benefits were quite modest (81). Thymic restoration is currently under investigation using embryonic stem cells and promising preclinical studies in rodents have demonstrated that the expansion of cTECs and mTECs, lead to increased numbers of functional T cells in the periphery (82).

Another approach to restore thymopoiesis is the use of postnatal allogeneic cultured thymus tissue, an approach that improved thymic output in patients with DiGeorge syndrome (83, 84). These patients are profoundly immunocompromised, which allow thymus engraftment without significant graft-rejection. Although such strategy has not been tested in allogeneic-SCT patients, it was investigated in HIV patients (85). However, high rate of thymus rejection was observed. Immunosuppression appears essential for successful thymic engraftment and T cell depletion prior to thymus transplant could lower the rate of graft rejection in GVHD patients. However, it is not clear whether lower numbers of CLPs observed after aGVHD could affect thymic output. Interestingly, IL-21 can improve thymopoiesis by expanding BM Lin^−^Sca1^+^c-kit^+^ lymphoid progenitors after allogeneic-SCT (86). Thus, combining thymus transplantation with either IL-21 therapy or T cell precursor therapy could yield better thymic recovery following allogeneic-SCT.

## Restoring the Peripheral Niche

Approaches to improve HP of mature lymphocytes after allogeneic-SCT have demonstrated that the benefit on T cell regeneration is frequently offset by excessive GVHD (4, 23, 87). IL-7 therapy has been shown to expand T cells with a predominant effect on naïve CD8^+^ cells (88). When administered early post-SCT, IL-7 could expand GVHD precursors and worsen aGVHD (4). At later time points, alloreactive T cells are activated and express lower IL-7Rα levels, which could explain why GVHD severity is less affected by IL-7 therapy (23, 89). Similarly, IL-15 can improve lymphocyte reconstitution after T cell depleted allogeneic-SCT (90), but it can also worsen GVHD (5, 91). DCs are the most potent cell type to initiate T cell activation and forcing their recovery following allogeneic-SCT can present significant risks. For instance, Flt3-L treatment can expand most DC subsets in mice and humans (92) and when administered after allogeneic-SCT, it worsens GVHD (23). In contrast, the expansion of DCs by SDF-1α therapy is largely restricted to the DC1 subset and resulted in a decrease in GVHD severity (23). Host DC1 have been shown to protect against aGVHD (93, 94). DC1 cells have an intrinsic ability to cross present antigens on MHC-I molecules and they are poor activators of CD4^+^ T cells (95, 96), a property that is likely important for preventing unwanted T cell activation during homeostatic stimulation of naïve lymphocytes. However, there are several different subsets of DC with distinct functions, and it is not clear which subset specifically controls CD4 homeostasis, if any (97). Thus, depending of the cocktail of cytokines used to improve T and DC regeneration, caution is needed as the risk to aggravate GVHD may surpass the benefit on T cell regeneration.

Mesenchymal stem cells (MSCs) can be administered in patients to diminish GVHD (98, 99). MSCs have immunosuppressive properties on T cells and on innate immune cells (100–102). MSCs produce a vast array of cytokines and growth factors known to affect hematopoiesis (103–107). While IL-7 production by MSCs may help to improve T cell survival, the secretion of other factors like SDF-1α could promote DC regeneration (108). The infusion of HLA mismatch MSCs was first reported to reduce alloreactivity in patients with steroid-refractory aGVHD (109). However, studies that follow showed a benefit highly variable and often transient with response rate higher in patients with mild GVHD (Grade-II) (110–112). MSC therapy can effectively induce complete response in 72% of patients (Grade II) with partial response for patients with higher grade GVHD (99, 113). Importantly, the use of MSCs generated from pooled BM mononuclear cells obtained from multiple allogeneic donors showed surprising efficacy in patients with grade III-IV refractory GVHD (114, 115). Thus, the use of multiple donors to generate MSCs could perhaps reduce the variability inherent to individual donor. The development of effective strategies to lower GVHD severity is clearly an important milestone in our attempt to restore T cell homeostasis. Such a reduction could diminish GVHD insults to the thymus and the peripheral niche, facilitating our future immune interventions to accelerate lymphocyte reconstitution in patients.

## Restoring T Cell Homeostasis after GVHD

In most clinical settings of human lymphopenia, the dysfunction of the thymus represents the primary limiting factor for T cell regeneration. However, the dysfunction of the peripheral niche is now well documented and added to the dysfunction of the thymus and together they contribute to the very poor immune reconstitution that typically occurs in GVHD patients. In a setting where the peripheral niche remains dysfunctional, it is not clear whether restoring thymopoiesis would be sufficient to increase peripheral T cell counts. DCs and IL-7 provide critical survival signals to RTEs for their expansion in the periphery. Depending of the robustness of the thymic output, T cell immune reconstitution may vary considerably when the peripheral niche is dysfunctional. The absence of peripheral DCs could result into greater competition between T cells for access to MHC class I–II, which could affect the number and the diversity of the T cell repertoire. Under these conditions, the repertoire may be skewed toward lymphocytes with the highest levels of self-reactivity. In addition, the absence of peripheral DCs could impede self-tolerance imposed by thymopoiesis since the peripheral DCs pick up peripheral self-antigens, migrate to the thymus and induce negative selection of developing self-reactive thymocytes (116, 117). The failure to present self-antigens through MHC-II has been shown to induce severe autoimmunity in mice transplanted with MHC-II^−/−^ BM cells (118). As a result, the feasibility and the safety of restoring thymopoiesis in GVHD patients remain largely unknown. Animal models used to study T cell homeostasis have not yet addressed the capacity of the thymus to restore T cell counts in the absence of a functional peripheral niche. The use of thymic transplantation in GVHD hosts could provide significant insights about the potential benefits and limitations of restoring thymopoiesis in GVHD patients.

## Conclusion and Perspectives

Despite significant progress in our understanding of the biology of T cell depletion, therapies aimed at accelerating T cell regeneration remain limited and are still in clinical development (119, 120). The effect of GVHD on T cell homeostasis is multiple and addressing the dysfunction of the thymus or the peripheral niche alone may yield disappointing results in patients undergoing allogeneic-SCT. Under these circumstances, the coordinate use of IL-7, SDF-1α, T cell precursors, embryonic stem cells, tissues engineering to recreate an artificial thymus and thymus transplantation could provide superior benefits with regards to T cell regeneration in GVHD patients. In parallel, we need to address the recovery of other immune cell subsets such as B- and/or NK cells, which also provide immunocompetence to transplanted patients. Collaboration between clinicians and scientists will be instrumental to the successful development of new animal models of thymic rejuvenation in GVHD hosts to address the dysfunction of peripheral niche in order to guide our future immune interventions to accelerate and restore the T cell compartment in humans.

## Author Contributions

All authors contributed intellectually to the writing of this work.

## Conflict of Interest Statement

The authors declare no commercial or financial relationships that could constitute a conflict of interest.
